# Catalytic Hairpin Assembly-Based Self-Ratiometric Gel Electrophoresis Detection Platform for Reliable Nucleic Acid Analysis

**DOI:** 10.3390/bios14050232

**Published:** 2024-05-07

**Authors:** Qiang Xi, Si-Yi Wang, Xiao-Bing Deng, Chong-Hua Zhang

**Affiliations:** 1 Hunan Prevention and Treatment Institute for Occupational Diseases, Affiliated Prevention and Treatment Institute for Occupational Diseases of University of South China, Changsha 410007, China; xiqiang@hnu.edu.cn (Q.X.); wangsiyi0066@163.com (S.-Y.W.); 2 School of Chemistry and Chemical Engineering, Hunan University of Science and Technology, Xiangtan 411201, China

**Keywords:** catalytic hairpin assembly, gel electrophoresis, ratiometric

## Abstract

The development of gel electrophoresis-based biodetection assays for point-of-care analysis are highly demanding. In this work, we proposed a ratiometric gel electrophoresis-based biosensing platform by employing catalytic hairpin assembly (CHA) process functions as both the signal output and the signal amplification module. Two types of nucleic acids, DNA and miRNA, are chosen for demonstration. The proposed strategy indeed provides a new paradigm for the design of a portable detection platform and may hold great potential for sensitive diagnoses.

## 1. Introduction

As a bioanalytical paradigm, nucleic acid analysis has great prospects in various applications in biology, chemistry, and clinical fields [1,2,3,4]. The polymerase chain reaction (PCR) remains to be the most widely used technique [5]. However, it is laborious, expensive, and requires complicated instruments, therefore its application is restricted in many resource-limited regions [2]. To circumvent these obstacles, many miniaturized analytical devices were developed, which proved to be portable and low cost, to provide rapid analysis, and to have a smaller intake of reagents and samples [6,7]. Despite the advances, they are often restricted by trade-offs between cost, sensitivity, and the ease of use. Thus, more efforts are needed in developing point-of-care nucleic acid analysis.

Gel electrophoresis is a workhorse of biological research since it was first reported 60 years ago, supplying a simple method to determine the topology, size, and quantity of RNA, DNA, and protein [8,9,10,11]. As a basic laboratory technique, gel electrophoresis displays the low-cost, accessible, and simultaneous analysis of multiple-component biomolecular interactions with a smaller sample volume. Recently, gel electrophoresis was employed in bioanalysis research to detect the reconfiguration of large DNA nanostructures, termed DNA nanoswitches [12,13,14,15]. The target-induced conformational change of DNA nanoswitches was applied in the detecting of miRNA [16,17], Zika virus [18], DNA [19,20,21], and more [22,23]. More recently, by combining the signal amplification feature of HCR with the CRISPR-Cas12a system, the Yigit group reported a reprogrammable gel electrophoresis-based approach for DNA virus detection [24]. The Zhang group developed a facile, multiple, and sensitive miRNA detection method based on conventional gel electrophoresis [25]. These reports highlight that gel electrophoresis is competitive in developing diagnostic assays. However, the absolute intensity of the gel bands is unavoidably disturbed by analyte-independent factors, such as the experimental conditions, biological samples, and human operations, which might limit the wider application of gel electrophoresis in diagnostic tests. Thus, the development of new gel electrophoresis-based chemo/biosensing to improve its accuracy and reliability for portable analysis is highly in demand.

Ratiometric assays are emerging as a powerful analytical technique, in which the target quantification is dependent on the ratio of two signals [26,27,28,29]. Thus, influences from instrumental or environmental factors could be effectively reduced, obtaining a more precise analysis than assays based on a single signal, especially for targets with low concentrations or in complex biological systems. Up to now, ratiometric analysis has been widely used for the quantitative detection of nucleic acids, proteins, and small molecules, most of which are based on fluorescence [30,31,32,33] and electrochemical [34,35] processes. Nevertheless, the design of gel electrophoresis-based ratiometric analysis assays, which enables the simultaneous realization of reliable, costless, and accessible analysis, are largely unexplored.

**Scheme 1:**
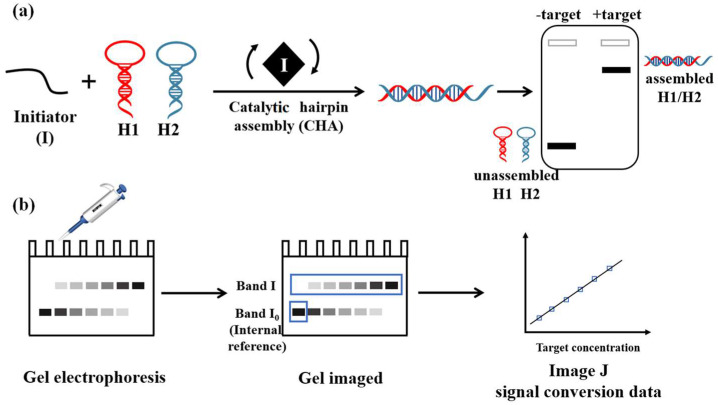
Illustration of the ratiometric gel electrophoresis assay. (**a**) Catalytic hairpin assembly; (**b**) The gel electrophoresis process and quantitative analysis by the band intensity.

Herein, we propose a ratiometric gel electrophoresis-based biosensing platform for accurate and reliable nucleic acid quantitative analysis, as illustrated in Scheme 1. In this system, a catalytic hairpin assembly process is employed to function as both the signal output and the signal amplification module. CHA has been widely used for sensing applications in virtue of its simple operation, mild conditions, and flexibility [36,37]. In this design, two substrate hairpin probes were designed, remaining unreactive in the absence of the target. Extra A bases were added on the 5′ of the oligonucleotide in H2 to ensure that Hl andH2 have the same migration rate in gel electrophoresis. When mixed with a sample containing the initiator I, these hairpins assembled stable duplexes with lager molecular weights. The assembled double-stranded products, which have lager molecular weights, can be distinguished simply with the use of substrate hairpins in gel electrophoresis due to the different migration rates. The DNA gel images are generated after gel electrophoresis, in which the appearance of a band I response, specifically to the initiator and the band intensity, is proportional to its concentration. The band intensity of the mixture of H1 and H2 is independent of the initiator concentration and is employed as an internal reference. Thus, the ratio of I/I_0_ is applied to evaluate the quantity of the initiator.

To our knowledge, this is the first time that a ratiometric gel electrophoresis biosensor has been developed while employing CHA as a signal output and signal amplification module. This development provides the following significant advantages: First, when compared to the PCR-based nucleic acid analysis methods, the gel electrophoresis-based analytical platform required miniaturized detection devices, used a smaller sample volume, required no strict requirements for the operators, and are costless. Second, the introduction of the internal reference reduced target-independent influences and offered a more precise analysis. Third, isothermal nucleic acid amplifications for nucleic acids can offer improved sensitivity without the thermocycling required in PCRs. Therefore, our ratiometric gel electrophoresis-based biosensor may provide a useful platform for accurate and reliable nucleic acid analysis in point-of-care applications.

## 2. Materials and Methods

### 2.1. Reagents and Materials

The DNA oligonucleotides were synthesized and purified through HPLC by Sangon Biotech (Shanghai) Co., Ltd. (Shanghai, China). The sequences of the miRNA and DNA are given in Table S1. The Endonuclease IV and 10×NEBuffer 3 (1000 mM NaCl, 500 mM Tris-HCl (pH 7.9), 100 mM MgCl_2_ and 10 mM DTT) were obtained from New England Biolabs (Ipswich, MA, USA). DSN and 10 × DSN master buffer (500 mM Tris-HCl, 50 mM MgCl_2_, 10 mM D, L-dithiothreitol (DTT), pH 8.0) were purchased from Evrogen (Moscow, Russia). The fetal bovine serum (FBS) was gained from Gibco (Carlsbad, CA, USA). The RNase inhibitor, DEPC-treated water, 10 × TBE buffer (225 mM Tris-Boric Acid, 50 mM EDTA, pH 8.0), 10 × phosphate-buffered saline (PBS), 4S green plus nucleic acid stain, and 10 × RNA glycerol gel loading buffer were obtained from Sangon Biological Engineering Technology & Services Co., Ltd. (Shanghai, China). Furthermore, 30% Acrylamide/bis-acrylamide, 29:1 (3.3% crosslinker), Ammonium Persulfate (APS), and N, N, N′, N′-Tetramethylethylenediamine (TEMED) were purchased from Bio-Rad Laboratories, Inc. (Hercules, CA, USA). Other chemicals were of analytical grade and were obtained from Sinopharm Chemical Reagents (Shanghai, China).

### 2.2. Catalytic Hairpin Assembly

All the hairpin probes were formed by heating the sample to 95 °C for 5 min, and then slowly cooling to 25 °C before use in PBS. Each of these DNA mixtures (1 μM of each hairpin and 100 nM of initiator I) were incubated in the reaction buffer (0.1 M PBS buffer, 1.37 M NaCl, 26.83 mM KCl, 81 mM Na_2_HPO_4_, 17.6 mM KH_2_PO_4_, pH = 7.4) at 37 °C for 3 h. Additionally, 20 μL of each sample was mixed with 2 μL of loading buffer and analyzed using 10% polyacrylamide gel electrophoresis (PAGE) in 1 × TBE solution at 100 V for 70 min. The mass ratio of acrylamide to N, N’-Methylene-bis(acrylamide) is 29:1. After they were stained with 4S green plus nucleic acid and water eluting, the resulting gel was imaged.

### 2.3. Gel Analysis

In ImageJ (version 1.54 h) and then clicking inside of each peak. All gels were stained using 4S green plus nucleic acid staining and water eluting three times; the gel images were captured using a Gel Documentation and Image Analysis System (ChampGel 7000). The exposure used for imaging was adjusted so that none of the bands were overexposed (indicated by the highlighted pixels). ImageJ was used for gel image analysis, and the gel image is first imported to ImageJ without any adjustments. Once imported, the image is converted to an 8-bit type, and each band of interest is highlighted using the rectangle tool. The plot for each lane was obtained using the ‘Analyze’ tool, and the numerical value for the peaks was obtained by selecting the wand tool.

### 2.4. Protocol for miRNA-21 and HBV Detection

The miRNA-21 was detected in a 20 μL amplification reaction mixture containing cDNA-21 (0.1 μM), 0.2 U DSN (solubilized in 25 mM Tris-HCl, pH 8.0, 50% glycerol), 0.8 U RNase inhibitor, 1 × DSN buffer, and different concentrations of target miRNA-21 at 65 °C for 30 min. Subsequently, the above solution was rapidly heated to 95 °C for 20 min in order to completely inactivate the DSN, and the final solution was slowly cooled down to 4 °C. Finally, this was followed by the addition of H1–21 (1 μM) and H2–21 (1 μM), which were both heated to 37 °C for 3 h.

### 2.5. HBV Detection

The HBV detection was performed in a 20 µL reaction mixture containing MB (0.1 μM), H1 (1 μM), H2 (1 μM), 0.3 U/μL endo IV, and different concentrations of target HBV (0 fM, 250 fM, 500 fM, 1 pM, 5 pM, 10 pM, 50 pM, 100 pM, 250 pM, 1 nM, 5 nM, 10 nM) in 1×NEBuffer 3 at 37 °C for 3 h. Furthermore, 2 μL of the loading buffer was added to all tubes. Additionally, 15 μL of each solution was loaded in a 10% polyacrylamide gel, and the electrophoresis was performed for 55 min at 100 V.

### 2.6. MiRNA-21 and HBV Quantification

MiRNA-21 and HBV quantification was achieved using PhotoMetrix^®^. Gels were stained using 4S green plus nucleic acid staining and water eluting three times, then the gel was placed in a Dark-box Type Ultraviolet Analysis Instrument (ZF-8); this was performed using a PhotoMetrix^®^ software version 1.2.1, obtained freely from the Play Store and installed on a smartphone (model MI 8, Xiaomi, Beijing, China). The method used to acquire the images is shown in Figure S1, and each band was quantified via the value of the captured image in a region of 32 × 32 pixels. The calibration curves were obtained via the insertion of the different concentrations of the target at each point, which were then used to calculate the amount of the target in the sample. Before the target analysis, preliminary tests were constructed to define the optimal distance between the camera and the gel electrophoresis (8 cm).

## 3. Results and Discussion

**Figure 1:**
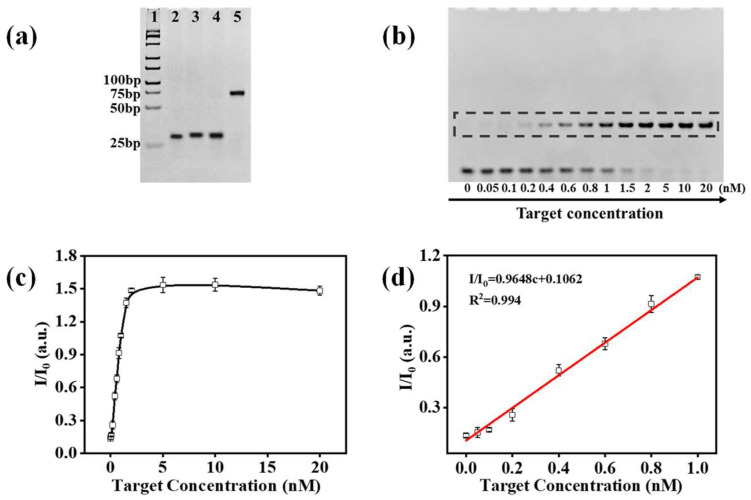
(**a**) Page gel electrophoresis images. Lane 1, Marker; Lane 2, H1; Lane 3, H2; Lane 4, H1 + H2; Lane 5, H1 + H2 + initiator; (**b**) Typical gel images of the target with varying concentrations; (**c**) Ratio of band intensity I/I_0_ versus the target concentrations; (**d**) Linear correlation between I/I_0_ and target concentrations range from 0 to 1 nM. Error bars are the standard deviations of three repetitive experiments. Concentrations of H1 and H2 are 100 nM.

A gel electrophoresis assay was constructed first by choosing an initiator strand as a model target to demonstrate the proof of principle. Two hairpin probes with equal bases were employed. As shown in Figure 1a, three clear bands were observed for H1, H2, and their mixture, respectively (lanes 2, 3, and 4). When incubating the two metastable DNA hairpins with the target, a new band of about 70 bp (lane 5) appeared, indicating the formation of the H1–H2 complex, a product of CHA reaction. The intensity of the double-stranded products in gel images were related to the target concentration (Figure S1); however, the reproducibility was too low for accurate quantitative analysis, with relative standard deviations as high as 32.8% across three parallel experiments in the presence of 10 nM of the initiator. This may be due to the band intensity in the gel images being unavoidably influenced by the image conditions and electrophoresis process. Interestingly, the intensity of the mixture of H1 and H2 would not be affected by the CHA reaction because no initiation sequence was added. After the imaging, the area of interest in each band was determined by the length and width of the control band, and the gray value of the blank sample was used as an internal reference. The ratio of I/I_0_ was supposed to eliminate non-specific interference in virtue of built-in corrections. Experimental results showed that, when the concentration of the target increased from 0 nM to 20 nM, the ratio of I/I_0_ was gradually enhanced, and a good linear relationship was obtained in a range from 50 pM to 1 nM with a much smaller standard deviation. The corresponding correlation coefficient (R^2^) was 0.994, with the limit of detection (LOD) estimated to be 300 pM (Figure 1d). These results revealed that the proposed ratiometric gel electrophoresis assay provided a reliable quantitative analysis platform.

**Figure 2:**
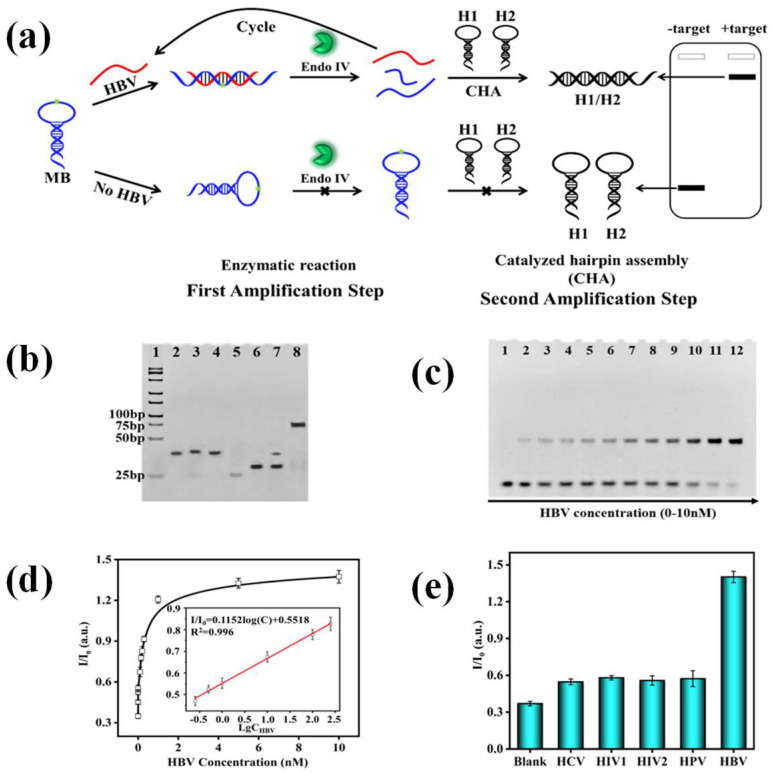
(**a**) Schematic illustration of the DARGE assay for HBV detection. (**b**) Typical gel eletrophoresis images of HBV analysis. Lane 1: Marker; Lane 2: MB; Lane 3: MB + HBV; Lane 4: MB+ Endo IV; Lane 5: MB + HBV + Endo IV; Lane 6: H1 + H2; Lane 7: MB + Endo IV + H1 + H2; Lane 8: MB+ HBV + Endo IV + H1 + H2. The concentration is 10 nM for HBV and MB, and 100 nM for H1 and H2, respectively. (**c**) Gel electrophoresis images of HBV with concentrations from 0 to 10 nM; (**d**) Ratio of band intensity I/I_0_ versus HBV concentrations. Inset: Linear correlation between I/I_0_ and target concentrations range from 0.25 pM to 250 pM. (**e**) The specificity evaluation of the DARGE strategy for HBV detection. The HBV concentration is 10 nM. Error bars are standard deviations of three repetitive experiments. I and I_0_ represent the band in the presence and absence of HBV, respectively.

After demonstrating that our ratiometric gel electrophoresis assay is feasible for the accurate analysis of specific target strands, we further studied if enzymatic amplification could be applied in the detection system to improve the sensitivity and diversify of the target type. First, hepatitis B virus (HBV) is chosen as a model DNA target (Figure 2). The helper probe MB contains a locked initiator sequence and a target complementary sequence with a tetrahydrofuran basic site (TAP site). When the target is present, the loop of MB would hybridize with HBV, and dsDNA products are formed. The TAP site in the dsDNA products can be recognized by the DNA repairing enzyme endo IV and can then be cleaved into two fragments, releasing the initiator for CHA amplification. The targets are then recycled, and more initiators are released. Thus, the CHA process is recycled and the ratio of I/I_0_ increased. However, in the absence of the target, the initiator is locked and can not trigger CHA, thus obtaining a low ratio of I/I_0_.

The feasibility of the dual amplification-based ratiometric gel electrophoresis analysis assay (DARGE) for HBV was first investigated. As shown in Figure 2b, MB remained intact in the absence of either HBV DNA or endo IV. When the targets were added into the mixture of CHA hairpins and the endo IV enzyme, an obvious double-strand product brand for CHA was observed. These results confirmed that the CHA reaction could be triggered by the HBV target in the detection system, thus meaning that validating the proposed design is viable for dual amplification-based gel electrophoresis analysis. We further optimized the experimental conditions for the better performance of the biosensor, including the reaction time and dosage of the enzyme (Figure S2).

Under optimized conditions, the self-ratiometric gel electrophoresis sensor was found to possess an increased ratio of I/I_0,_ dynamically correlated to the concentrations of HBV (Figure 2c,d). DARGE permitted the detection of HBV from a 10 μL sample, with concentrations ranging over four orders of magnitude with an LOD of 130 fM. This detection limit was much better than CHA alone-based gel electrophoresis analysis, indicating the advantage of sensitivity enhancement for our isothermal dual amplification system. Next, the specificity of the proposed assay for HBV was tested. The target HBV led to the enhancement of the band intensity of the CHA product, with an I/I_0_ ratio ∼1.4-fold increase (Figure 2e), while, less drastically, enhancements were observed in response to the following four virus DNA sequences: HCV, HIV1, HIV2, and HPV. This result validated the specificity of the proposed gel electrophoresis system for HBV analysis. To invest the DARGE strategy in complex biological media, we further detected HBV in 10% human serum samples. Satisfactory recoveries between 96.6%–107.2% were obtained by adding HBV in three different concentrations into 10% human serum (Table S2, ESI), indicating the potential of the DAGRE assay for real sample analysis.

Smartphones have attracted a lot of attention for on-site detection, owing to its low cost and simple setup and operation. We further employed a smartphone camera to acquire the gel image, which was further analyzed using PhotoMetrix^®^ app. The operating processes are illustrated in Figure S3. The data analysis was constructed as shown in Figure S4 using the PhotoMetrix^®^ app. A quasilinear correlation curve was obtained, ranging from 1 pM to 5 pM with R^2^ 0.992 (Figure S5) with satisfying recoveries (Table S3). These results demonstrated that this developed smart phone-combined ratiometric gel electrophoresis platform is promising for applications in portable analysis.

**Figure 3:**
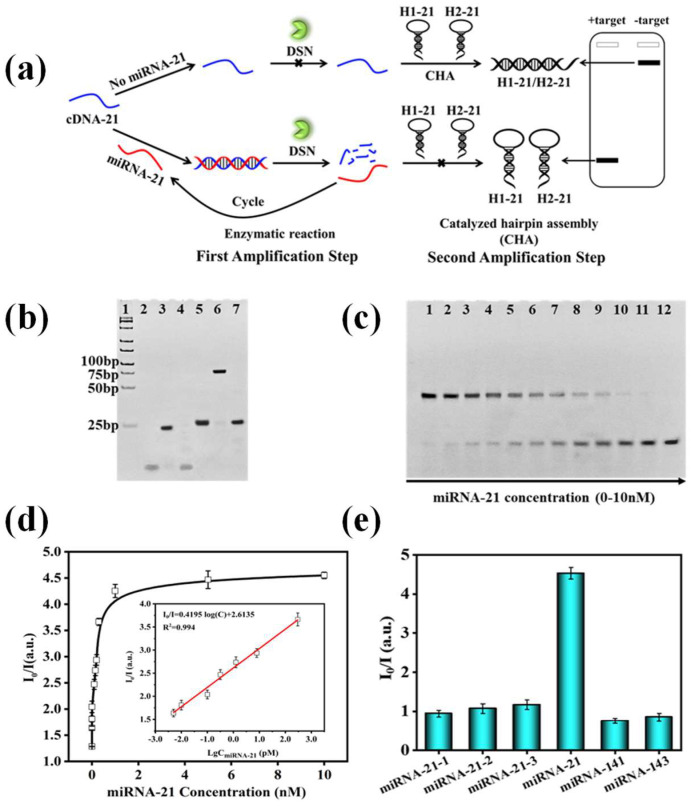
(**a**) Schematic representation of miRNA detection based on the DSN−assisted gel electrophoresis assay. (**b**) Typical gel electrophoresis images of miRNA−21 analysis. Lane 1: Marker; Lane 2: cDNA−21; Lane 3: cDNA−21 + miRNA 21; Lane 4: cDNA−21 + miRNA 21 + DSN; Lane 5: H1 + H2; Lane 6: H1 + H2 + cDNA−21; Lane 7: H1 + H2 + cDNA−21 + miRNA 21 + DSN. The concentration is 10 nM for cDNA−21, miRNA 21 MB, and 100 nM for H1 and H2, respectively. (**c**) Gel electrophoresis images of miRNA with varying concentrations: 1–12.0 nM, 0.2 × 10^−3^ nM, 0.5 × 10^−3^ nM, 1 × 10^−3^ nM, 1 × 10^−2^ nM, 5 × 10^−2^ nM, 1 × 10^−1^ nM, 3 × 10^−1^ nM, 5 × 10^−1^ nM, 1 nM, 5 nM, and 10 nM, respectively; (**d**) Ratio of I/I_0_ versus miRNA concentrations. Inset: Linear correlation between I/I_0_ and target concentrations range from 200 fM to 300 pM. (**e**) The selectivity of the proposed method via assessing different miRNA targets. The concentration of each miRNA sample is 10 nM. I and I_0_ represent the band in the presence and absence of miRNA, respectively. Error bars are standard deviations of three repetitive experiments.

The developed assay was further employed for miRNA detection to demonstrate its generality, in which duplex-specific nuclease signal amplification was employed to improve the specificity and sensitivity. The principle of the design was shown in Figure 3a. A c-DNA was designed as the initiator for CHA, which was completely complementary to the target miRNA. Upon the addition of the target, the ssDNA probe hybridizes to a target miRNA to form a DNA/RNA heteroduplex, which will be the substrate for DSN cleavage. The cleaved c-DNA could not initiate CHA, thus no CHA double-stranded product was formed. The cleavage of the c-DNA also leads to the target miRNA being released and a next round of cleavage being initiated. However, if the targets are absent, the c-DNA remains active and enables the formation of CHA products. To make a turn-on assay, the ratio of the band intensity I_0_/I was applied to indicate the target concentration. MiRNA-21 was chosen as a model target, which is overexpressed in human breast cancers [38], to demonstrate the feasibility of the developed strategy.

As shown in Figure 3b, in the presence of the target miRNA, the band of c-DNA almost entirely disappeared (lane 4), which could be ascribed to the high digestion efficiency of the DSN to DNA in the DNA/RNA heteroduplex. In the absence of the target, the product band intensity increased, accompanied with a diminished hairpin band intensity, regardless of the addition of DSN (lane 6), indicating the occurrence of CHA and the consumption of the hairpins. However, after the addition of the target, a sparce consumption of hairpins was observed. As shown in Figure 3c, the increasing of the hairpin band intensity was positively related to the miRNA concentration. Under optimized conditions (Figure S6), a quasilinear correlation was obtained for the ratio of I_0_/I to the logarithmic miRNA concentrations, ranging from 200 fM to 300 pM, with the detection limit estimated to be 150 fM (Figure 3d), with satisfying recoveries in 10% human serum. As little as 1.5 amol of the target could be detected with a sample volume of 10 μL, indicating the high sensitivity of the proposed method. Satisfying specificity for the target miRNA detection was further acquired (Figure 3e). A quasilinear correlation was obtained for the ratio of I_0_/I to the logarithmic miRNA concentrations in the range of 0.2 pM–1.5 pM with R^2^ 0.993 (Figure S7), using the smart phone-combined DARGE assay with an ideal recovery (Table S5).

## 4. Conclusions

In conclusion, we developed a novel ratiometric gel electrophoresis biosensing platform, employing dual isothermal amplification for accurate and reliable nucleic acid quantitative analysis. To our knowledge, this is the first time a catalytic hairpin assembly process has been introduced in ratiometric gel electrophoresis biosensor functions as a signal output and amplification module. The self-ratiometric biosensor provided advantages of miniaturized instruments, simple operation, and high reproducibility. Moreover, dual isothermal amplifications for nucleic acids enable improved sensitivity at a smaller cost. Two types of nucleic acids, DNA and miRNA, are chosen for demonstration. The proposed assay shows the detection limit to be 130 fM for HBV and 150 fM for miRNA 21, respectively. The developed method also displays ideal selectivity against nonspecific interference. Satisfying recoveries were obtained in the serum sample, implying the great potential of the ratiometric gel electrophoresis strategy for real sample analysis. More interestingly, this developed assay also has the potential to enable rapid point-of-care detection, as we demonstrate when performing DARGE using a smartphone camera for imaging. Moreover, this strategy also has the potential to be extended for small molecule and protein detection by combining aptamer recognition. Owing to these advantages, the developed ratiometric gel electrophoresis strategy indeed provides a new paradigm for the design of portable detection platforms, and might hold great potential for sensitive diagnosis.

## Data Availability

The original data and contributions presented in this study are included within the article or Supplementary Materials. For further inquiries, please contact the corresponding author.

## Supplementary Materials

The following supporting information can be downloaded at: https://www.mdpi.com/article/10.3390/bios14050232/s1, Scheme S1: Schematic illustration of catalytic hairpin assembly; Table S1: Oligonucleotides sequences used in the experiment; Table S2: Recovery experiments for HBV detection in 10% human serum; Table S3: Smart phone-combined DARGE assay for HBV detection; Table S4: Recovery experiments for miRNA-21 detection in 10% human serum; Table S5: Smart phone-combined DARGE assay for miRNA-21 detection; Figure S1: CHA-based non ratiometric gel electrophoresis analysis; Figure S2: Optimization of experimental conditions on analytical performance in HBV assay; Figure S3: Schematic diagram of smartphone-based gel electrophoresis analysis for quantifying specific target; Figure S4: PhotoMetrix® application interfaces; Figure S5: Smart phone-combined DARGE ASSAY for HBV detection; Figure S6: Optimization of experimental conditions on analytical performance in miRNA assay; Figure S7: Smart phone-combined DARGE assay for HBV detection.
