# Supplementary material for: Catalytic Hairpin Assembly-Based Self-Ratiometric Gel Electrophoresis Detection Platform for Reliable Nucleic Acid Analysis
Source: Biosensors (Basel). 2024 May 7;14(5):232. doi: 10.3390/bios14050232 (PMC11118829; doi:10.3390/bios14050232)
Supplement: Supplementary file 1 [file biosensors-14-00232-s001.zip › biosensors-2969373-supplementary.pdf]

Communication

Catalytic Hairpin Assembly-Based Self-Ratiometric Gel Electrophoresis Detection Platform for Reliable Nucleic Acid Analysis

Qiang Xi [1], Si-Yi Wang [1], Xiao-Bing Deng [1,*] and Chong-Hua Zhang [2,*]

[1] Hunan Prevention and Treatment Institute for Occupational Diseases, Affiliated Prevention and Treatment Institute for Occupational Diseases of University of South China, Changsha 410007, China; xiqiang@hnu.edu.cn (Q.X.); wangsiyi0066@163.com (S.-Y.W.)

[2] School of Chemistry and Chemical Engineering, Hunan University of Science and Technology, Xiangtan 411201, China

* Correspondence: xdyyyjk@163.com (X.-B.D.); chonghua1226@hnu.edu.cn (C.-H.Z.)

Table of Contents

S-2 Scheme S1. Schematic illustration of catalytic hairpin assembly.

S-3. Table S1. Oligonucleotides sequences used in the experiment.

S-4. Table S2. Recovery experiments for HBV detection in 10% human serum.

S-5. Table S3. Smart phone-combined DARGE assay for HBV detection.

S-6. Table S4. Recovery experiments for miRNA-21 detection in10% human serum.

S-7. Table S5. Smart phone-combined DARGE assay for miRNA-21 detection.

S-8. Figure S1. CHA-based non ratiometric gel electrophoresis analysis.

S-9. Figure S2. Optimization of experimental conditions on analytical performance in HBV assay.

S-10. Figure S3. Schematic diagram of smartphone-based gel electrophoresis analysis for quantifying specific target.

S-11. Figure S4. PhotoMetrix® application interfaces.

S-12. Figure S5. Smart phone-combined DARGE ASSAY for HBV detection.

S-13. Figure S6. Optimization of experimental conditions on analytical performance in miRNA assay.

S-14. Figure S7. Smart phone-combined DARGE assay for HBV detection.

Scheme S1. Schematic illustration of catalytic hairpin assembly

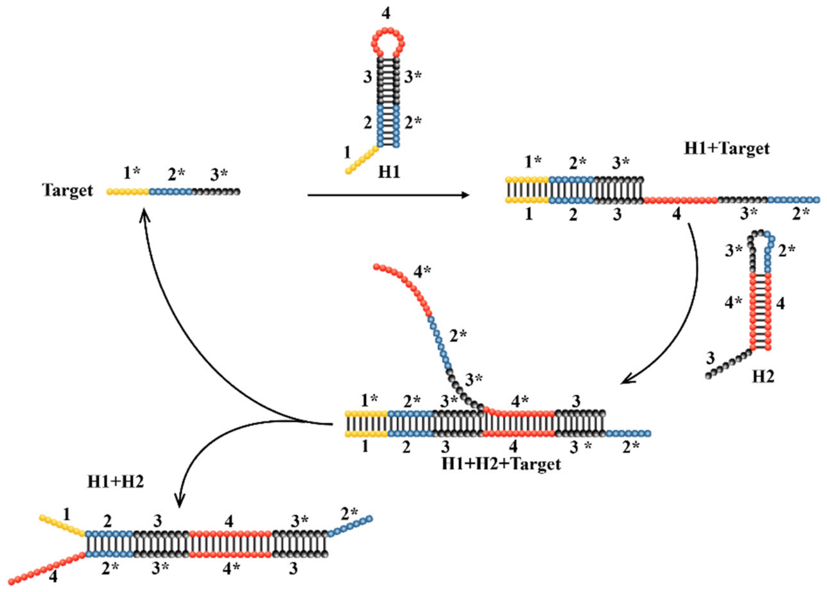

Table S1. Oligonucleotides sequences used in the experiment

Name	Sequence (5′-3′)
I	CCCAGGTTAACTTAGCTCACTGAC
H1	GTCAGTGAGCTAAGTTAACCTGGGCCATGAGAAGAC<br>CCAGGTTAACTTAGC
H2	AAAAAACCTGGGTCTTCTCATGGCCCAGGTTAACTTA<br>GCCCATGAGAAGAC
miRNA-141	UAACACUGUCUGGUAAAGAUGG
miRNA-143	UGAGAUGAAGCACUGUAGCUCA
miRNA-21	UAGCUUAUCAGACUGAUGUUGA
miRNA-21-1	UAGCUUAUCAGACUGAUCUUGA
miRNA-21-2	UAGCUUAUCAGACUAAUGUUGA
miRNA-21-3	UAGCUUAUCATACUGAUGUUGA
cDNA-21	TCAACATCAGTCTGATAAGCTA
H1-21	TAGCTTATCAGACTGATGTTGACCATGAGAAGATCA<br>ACATCAGTCTGA
H2-21	AAGATGTTGATCTTCTCATGGTCAACATCAGTCTGA<br>CCATGAGAAGAC
HBV	TTGGCTTTCAGTTATATGGATGATGTGGTA
MB	CCCAGGTTAACTTAGCTCACTGACTCCATATA<br>ACT**X**(AP)GAAAGCCAAGTCAGTGAGCTAA

* The underlined bases in miR-21s are mismatched bases. X represents abasic sites.

Table S2. Recovery experiments for HBV detection in 10% human serum

samples	spiked	found	RSD (%, n=3)	Recovery (%)
10% human serum	1.000 pM	1.072 pM	5.35	107.2
	5.000 pM	2.415 pM	3.29	96.6
	10.000 pM	3.163 pM	1.61	105.4

Table S3. Smart phone-combined DARGE assay for HBV detection

samples	spiked	found	RSD (%, n=3)	Recovery (%)
PBS	1.000 pM	1.083 pM	4.42	108.3
	2.500 pM	2.385 pM	3.53	95.4
	3.000 pM	3.174 pM	1.36	105.8

Table S4. Recovery experiments for miRNA-21 detection in10% human serum

**samples**	**spiked**	**found**	**RSD (%, n=3)**	**Recovery (%)**
10% human serum	1.000 pM	0.936 pM	3.81	93.6
	5.000 pM	5.415 pM	1.62	108.3
	10.000 pM	9.435 pM	2.37	94.4

Table S5 Smart phone-combined DARGE assay for miRNA-21 detection

samples	spiked	found	RSD (%, n=3)	Recovery (%)
PBS	0.700 pM	0.680 pM	3.73	97.1
	1.000 pM	1.024 pM	1.53	102.4
	1.500 pM	1.423 pM	2.29	94.8

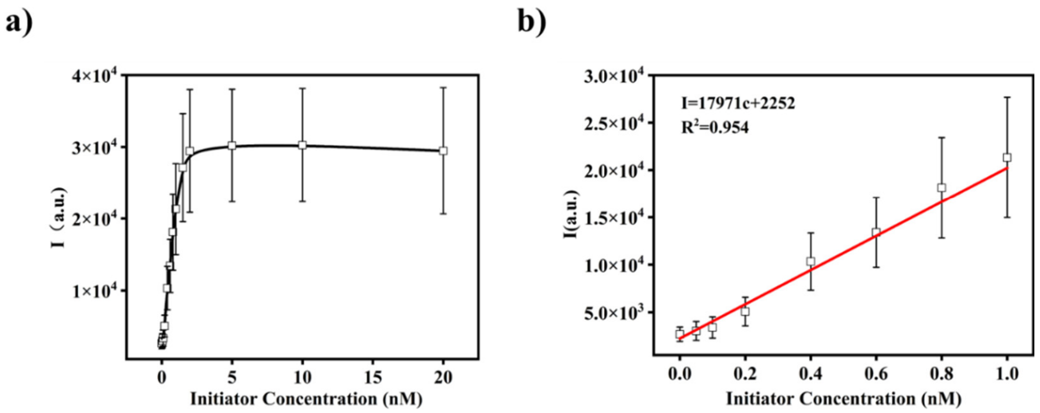

Figure S1. CHA-based non ratiometric gel electrophoresis analysis: (a) Band intensity versus target concentrations. (b) Linear correlation between I and target concentrations range from 0 to 1 nM. Error bars are standard deviations of three repetitive experiments.

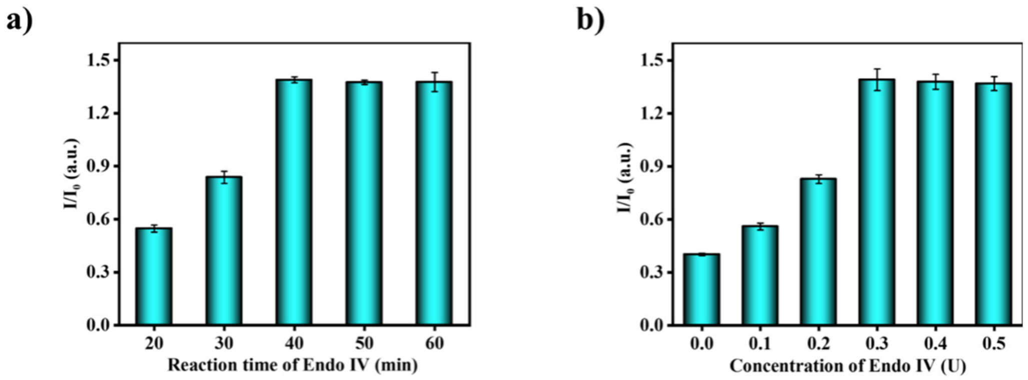

Figure S2. Optimization of experimental conditions on analytical performance. (a) Endo IV reaction time. (b) The amounts of Endo IV.

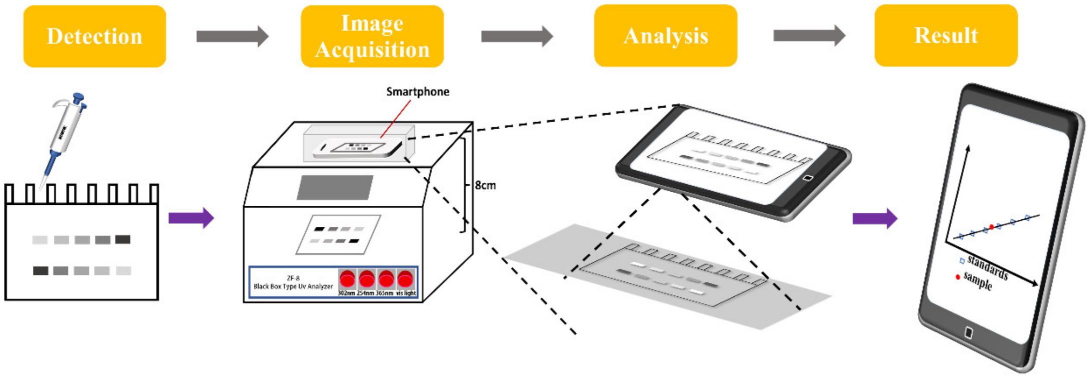

Figure S3. Schematic diagram of smartphone-based gel electrophoresis analysis for quantifying specific target.

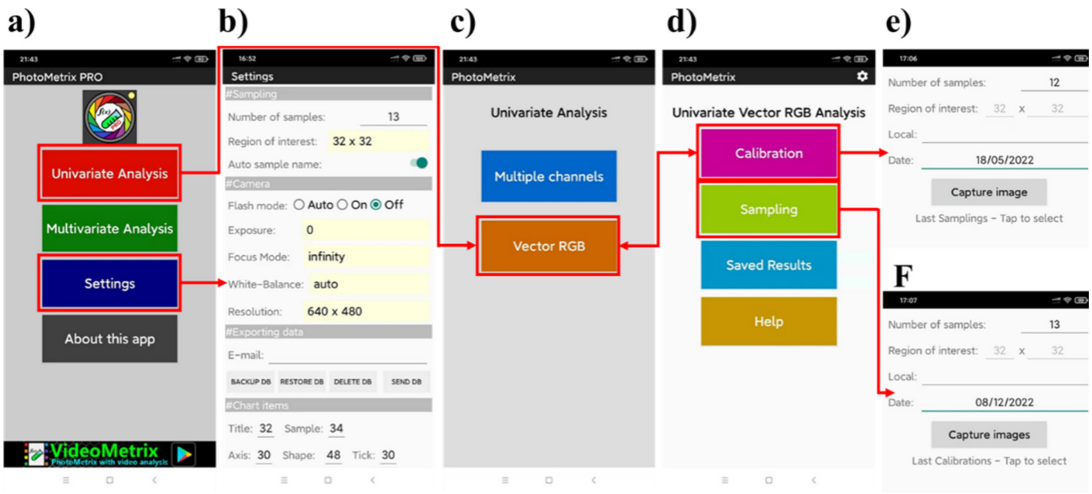

Figure S4. PhotoMetrix® application interfaces: (a) Home screen. (b) Settings screen. (c) Choice of color model for univariate analysis. (d) Interface for univariate analysis. (e) Build the calibration curves and (f) Sampling.

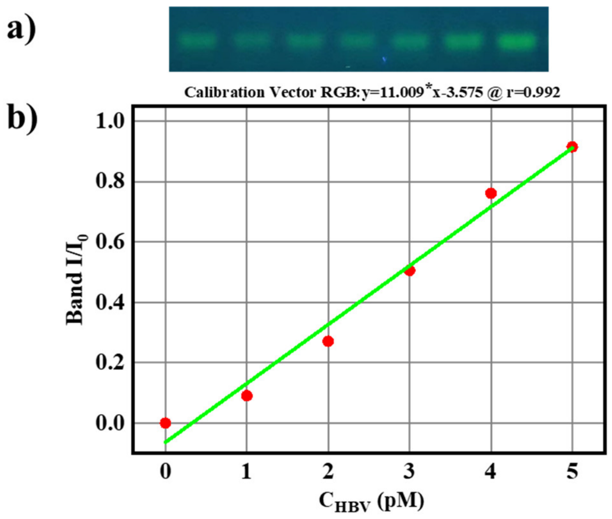

Figure S5. (a) Gel image acquired by smart phone camera. (b): Linear correlation between I/I0 and HBV concentrations range from 0 pM to 5 pM acquired by PhotoMetrix® software.

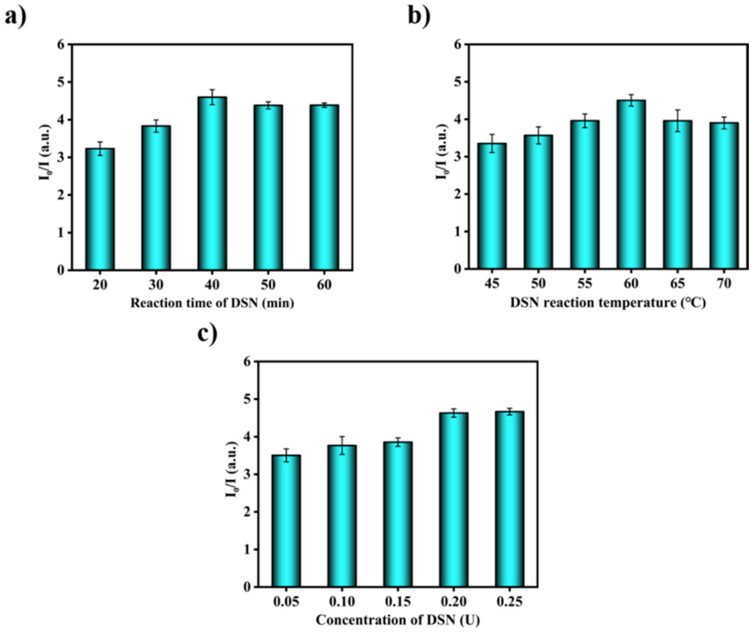

Figure S6. Optimization of experimental conditions on analytical performance. (a) The reaction time of DSN cleavage double-stranded. (b) Reaction temperatures of DSN. (c) The amount of DSN.

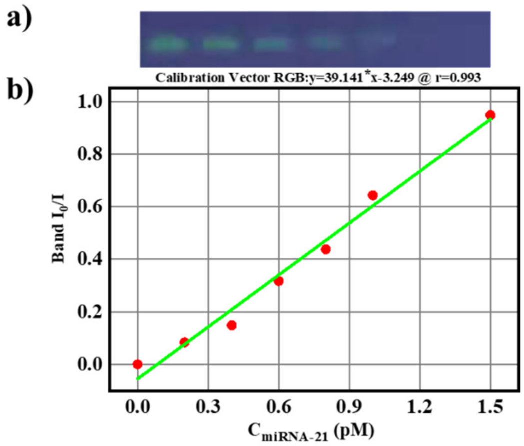

Figure S7. (a) Gel image acquired by smart phone camera for miRNA assay. (b) Linear correlation between I/I0 and miRNA concentrations range from 0 pM to 1.5 pM acquired by PhotoMetrix® software.
